# ER stress inhibitor attenuates hearing loss and hair cell death in *Cdh23*^*erl/erl*^ mutant mice

**DOI:** 10.1038/cddis.2016.386

**Published:** 2016-11-24

**Authors:** Juan Hu, Bo Li, Luke Apisa, Heping Yu, Shami Entenman, Min Xu, Ruben Stepanyan, Bo-Jhih Guan, Ulrich Müller, Maria Hatzoglou, Qing Yin Zheng

**Affiliations:** 1 Transformative Otology and Neuroscience Center, Binzhou Medical University, Yantai, Shandong, China; 2 Department of Otorhinolaryngology-HNS, Second Affiliated Hospital, Xi'an Jiaotong University School of Medicine, Xi'an, Shaanxi, China; 3 Department of Otolaryngology-HNS, Case Western Reserve University, Cleveland, OH, USA; 4 Department of Neurosciences, Case Western Reserve University, Cleveland, OH, USA; 5 Department of Genetics and Genome Sciences, Case Western Reserve University, Cleveland, OH, USA; 6 Department of Molecular and Cellular Neuroscience, Dorris Neuroscience Center, The Scripps Research Institute, La Jolla, CA, USA

## Abstract

Hearing loss is one of the most common sensory impairments in humans. Mouse mutant models helped us to better understand the mechanisms of hearing loss. Recently, we have discovered that the erlong (erl) mutation of the cadherin23 (*Cdh*23) gene leads to hearing loss due to hair cell apoptosis. In this study, we aimed to reveal the molecular pathways upstream to apoptosis in hair cells to exploit more effective therapeutics than an anti-apoptosis strategy. Our results suggest that endoplasmic reticulum (ER) stress is the earliest molecular event leading to the apoptosis of hair cells and hearing loss in *erl* mice. We also report that the ER stress inhibitor, Salubrinal (Sal), could delay the progression of hearing loss and preserve hair cells. Our results provide evidence that therapies targeting signaling pathways in ER stress development prevent hair cell apoptosis at an early stage and lead to better outcomes than those targeting downstream factors, such as tip-link degeneration and apoptosis.

The cadherin 23 (CDH23) protein is localized in the upper part of the tip link in hair cells, where it functions as a key component.^1, 2, 3^ In humans, *Cdh*23 mutations cause nonsyndromic autosomal recessive deafness (DFNB12) and Usher syndrome type 1D, USH1D, characterized by deafness associated with retinitis pigmentosa and vestibular dysfunction.^4, 5, 6^ In mice, *Cdh*23 mutations lead to hearing loss with or without vestibular dysfunction.^7, 8, 9^ Recently, we identified a novel point mutation (T208C) of *Cdh*23 and named this mutation erlong (*erl*).^10^ The *Cdh23*^*erl/erl*^ mice (*erl* mice) proved to be animal models of DFNB12. Previously, we showed that hair cell apoptosis is one of the pathological mechanisms leading to hearing loss in this mutant.^10^ Here, we aimed to reveal that ER stress signaling is the upstream pathway leading to hair cell apoptosis in mice with the *erl* mutation, and we sought to find potential therapeutics.

The perturbation of endoplasmic reticulum (ER) homeostasis leads to the accumulation of unfolded or misfolded proteins in the ER lumen, resulting in ER stress. The unfolded protein response (UPR) is subsequently triggered to alleviate this stress and to restore ER homeostasis, promoting cell adaptation and survival. Conversely, if the stress is prolonged, or if the adaptive response fails, the apoptosis pathway will be initiated.^11, 12, 13^ The UPR is mediated through three ER transmembrane receptors: protein kinase RNA-like ER kinase (PERK); activating transcription factor-6 (ATF6); and inositol-requiring enzyme 1 (IRE1).^11, 12, 13, 14^ Upstream of this network, the ER chaperone immunoglobulin-binding protein (BiP) functions as a main regulator by dissociating from the PERK, ATF6 and IRE1 luminal domains and thus activating these effectors' stress responses. As a downstream signal, the CCAAT/enhancer-binding protein-homologous protein (CHOP) is considered to be a very sensitive indicator of ER stress conditions, and its induction mostly promotes cell death.^11, 13^

A recent study of zebrafish mutants revealed that the Usher proteins, including CDH23, formed a complex and then preassembled at the ER.^15^ Defects in the Usher proteins disrupted the complex formation, induced the protein-trafficking defects, and finally triggered ER stress and apoptosis. Hence, blocking ER stress pro-apoptosis factors could reduce cell death in zebrafish mutants.^15^ Because the *erl* mutation affects *Cdh*23, the same gene that causes USH1D in humans, it is reasonable to speculate that the ERL-CDH23 protein might endure defective trafficking, thus triggering ER stress and leading to hair cell apoptosis and hearing impairment in *erl* mutants. In the current study, we tested the expression of the ER chaperone BiP and the pro-apoptotic factor CHOP in the *erl* and control C57BL/6 J (B6) mouse cochleae. Our data show that ER stress and the UPR response were the underlying causes of apoptosis in the *erl* cochleae, with the PERK signaling being a contributor. More importantly, we report here that the small molecular compound of ER stress inhibitor Salubrinal (Sal) could slow down the progression of hearing loss and hair cell death in *erl* mice. These results offer a potential therapy for human DFNB12 and possibly protect the visual function of individuals with Usher syndrome.

## Results

### CDH23 partly failed to reach the top of hair bundles and were co-localized with BiP in the subapical regions of OHCs in *erl* mice

**Figure 1:**
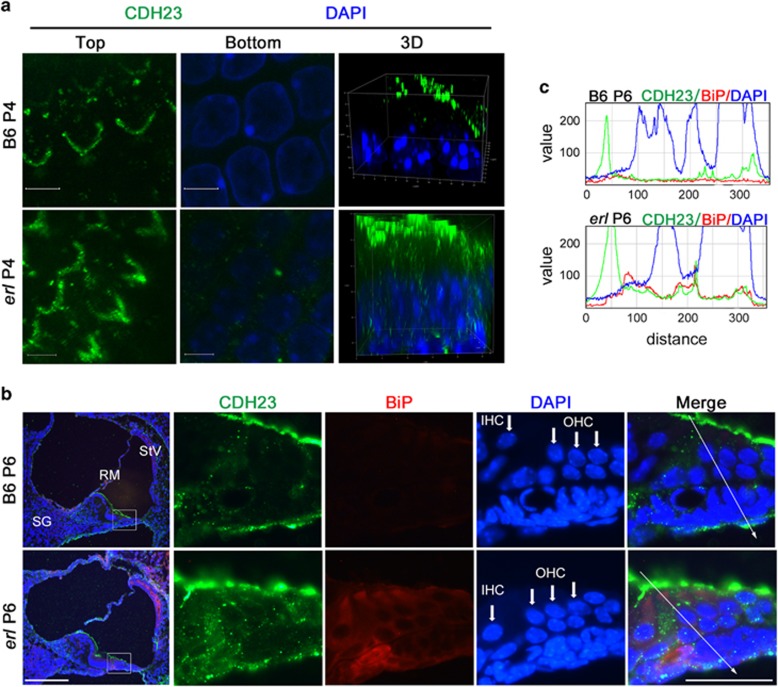
CDH23 expression and co-localization with BiP in *erl* mouse cochleae. (**a**) Top and bottom confocal stack sections and 3D reconstruction of confocal images (x63) revealed the location of CDH23 in the B6 (voxel-size 512 × 512 × 0.045 *μ*m^3^ and total volume 23.2 × 23.2 × 14.9 *μ*m^3^) and *erl* (voxel-size 1024 × 1024 × 0.027 *μ*m^3^ and total volume 27.2 × 27.2 × 27.2 *μ*m^3^) OHCs at P4. In the B6 mice, CDH23 protein was specifically localized at the tip of stereocilia of OHCs. The *erl* mice OHCs showed a wide range of CDH23 expressions (from the stereocilia to the nucleus). Around the nuclei, no detectable CDH23 signal was found in B6 mice, while in *erl* mice, many spots of CDH23 signal was detected in this region (*n*=3 mice per group). Scale bars, 5*μ*m. (**b**) The location and co-localization of BiP and CDH23 in the B6 and *erl* cochleae at P6. The B6 mice cochleae showed faint BiP signals and clear CDH23 signals, particularly on the tops of the OHCs. BiP was highly detected in the OHCs, SG, and StV of *erl* mice, and it was co-localized with CDH23 in the subapical regions of the OHCs (*n*=3 mice per group). Scale bars, 50 *μ*m (left); 20 *μ*m (right). (**c**) The line charts show two-dimensional graphs of the raw fluorescence intensities of the white arrows drawn in the panels to the left, as analyzed by Image J software. The *X*-axis represents distance (pixels) along the line, and the *Y*-axis is the pixel intensity. The BiP signals were highly expressed in *erl* mice and partially overlapped with the CDH23 signals (*n*=3 mice per group)

We measured the CDH23 distribution in cochlear outer hair cells (OHCs) in *erl* mice and B6 mice. At P4, the CDH23 protein was specifically localized at the top of the OHCs in B6 mice, as revealed by confocal 3D images, indicating the localization of the tip links in hair bundle stereocilia (Figure 1a). In contrast, the CDH23 protein localized from the stereocilia to the nuclei of the OHCs in *erl* mice. Portions of the CDH23 proteins failed to reach the top of the hair bundles and remained in the OHC cytoplasm. Immunostaining showed that the ER chaperone BiP was more readily detected in almost the same region and co-localized with cytoplasmic CDH23 in the *erl* mouse OHCs at P6 (Figure 1b), whereas no specific BiP signaling was detectable in the cytoplasm of the B6 mouse OHCs. Line charts confirmed the higher expression of BiP and its co-localization with CDH23 in the *erl* OHC cytoplasm (Figure 1c) when compared with the fluorescence intensity of CDH23 and BiP.

### The PERK arm of UPR was activated in the *erl* mouse cochleae

**Figure 2:**
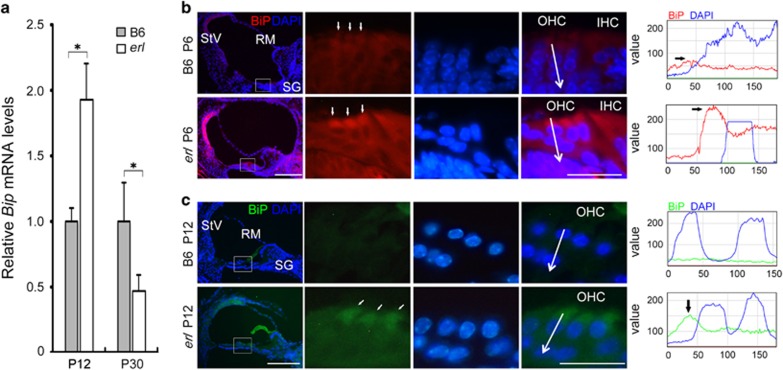
BiP expression in the B6 and erl mouse cochleae. (**a**) The *Bip* mRNA levels in the cochleae of the B6 and *erl* mice at P12 and P30. The *Bip* mRNA level was significantly higher in the *erl* mice at P12 but decreased at P30, compared with the B6 cochleae. The error bars represent S.E.M. (*P<0.05, *n*=3 mice, *t*-test). (**b**,**c**) The BiP showed higher intensities in the StV, SG and especially OHCs of the *erl* mice, forming a hat-like pattern on the top of the OHCs' nuclei at P6 and P12. A line intensity analysis revealed that the BiP signals were expressed in the OHC area of the *erl* cochleae (*n*=4 mice per group). Scale bars, 50 *μ*m (left); 20 *μ*m (right)

**Figure 3:**
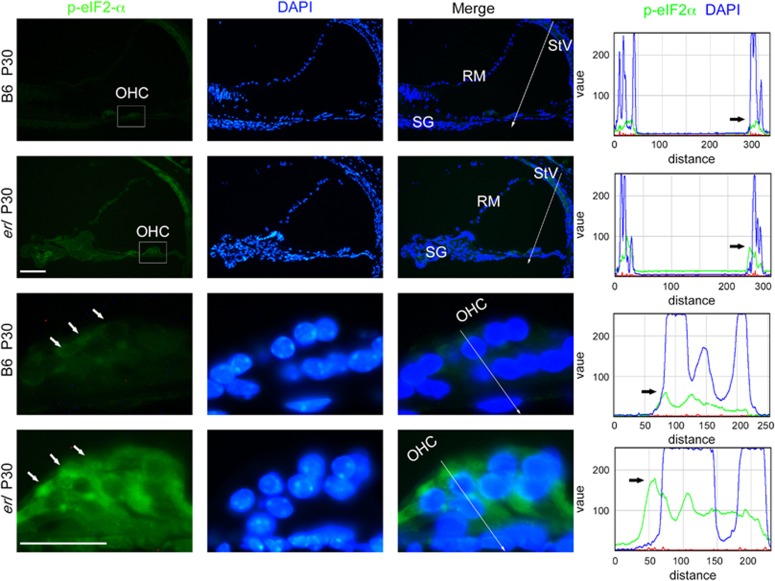
The eIF2α phosphorylation in the B6 and *erl* cochleae at P30**.** The immunefluorescence labeling of phospho-eIF2α showed strong cytoplasmic and perinuclear localization in the cochleae of the *erl* mice. The line intensity analysis along the arrow shown in the panels to the left revealed that the p-eIF2α signals were expressed in the OHC and stria vascularis (StV) areas and were strongly detected around the nuclei in the *erl* cochleae (*n*=3 mice per group). Scale bars, 50 *μ*m (upper); 20 *μ*m (lower)

The ER stress marker BiP was upregulated in the *erl* mice inner ears, as compared with those of B6 mice. In the measurements of mRNA isolated from the *erl* mouse cochleae, BiP was upregulated at P12 but downregulated at P30 (Figure 2a). Immunostaining showed not only a greater expression of BiP in OHCs but also that BiP was expressed at higher levels in spiral ganglion (SG) cells and in stria vascularis (StV) in the *erl* cochleae at P6 and P12 (Figure 2b and c). Consistent with the confocal three-dimensional (3D)_images, the BiP signals were mainly localized in the OHC cytoplasm and formed a hat-like pattern above the OHCs' nuclei (Figure 2b and c). ER stress effector PERK's kinase target, the phosphorylated alpha-subunit of the eukaryotic initiation factor (eIF2α), also showed strong localization in the perinuclear regions of the OHCs in the *erl* cochleae (Figure 3).

**Figure 4:**
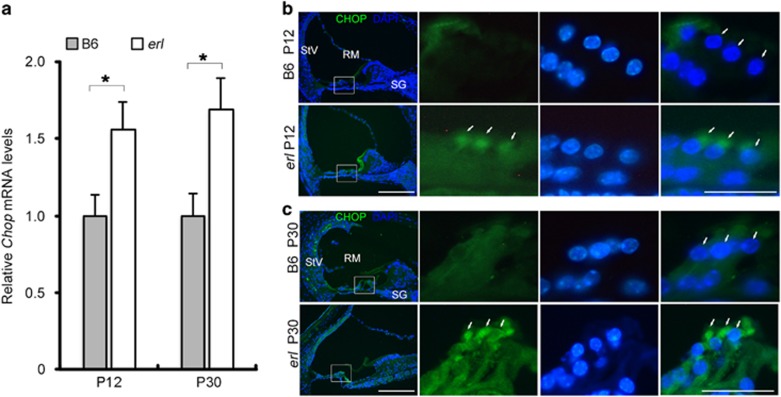
CHOP expression in the B6 and *erl* mouse cochleae. (**a**) The *Chop* mRNA levels in the cochleae of the B6 and *erl* mice at P12 and P30. The *Chop* mRNA level was significantly higher at P12 and P30 in the *erl* cochleae than in the B6 cochleae. The error bars represent S.E.M. (*P<0.05, *n*=3 mice, *t-*test). (**b**,**c**) CHOP maintained greater intensities in in stria vascularis (StV), spiral ganglion (SG), especially in the perinuclear region between nucleus and hair bundles of the OHCs in the *erl* mice at P12 and P30 (*n*=3 mice per group). Scale bars, 50 *μ*m (left); 20 *μ*m (right)

Downstream of the PERK arm, the pro-apoptotic factor CHOP was also expressed at higher levels in the *erl* cochleae (Figure 4). At the mRNA level, Chop maintained a higher expression in the *erl* cochleae compared with B6 cochleae at P12 and P30 (Figure 4a). Immunostaining performed at the same age showed that CHOP was highly detected in the OHCs, SG and StV of *erl* mice and was mainly detected in the perinuclear regions of OHCs (Figure 4b and c). However, a bare CHOP signal was present in B6 mouse cochleae.

### Disruption of the *Chop* gene protected hearing and OHCs in *erl* mice

**Figure 5:**
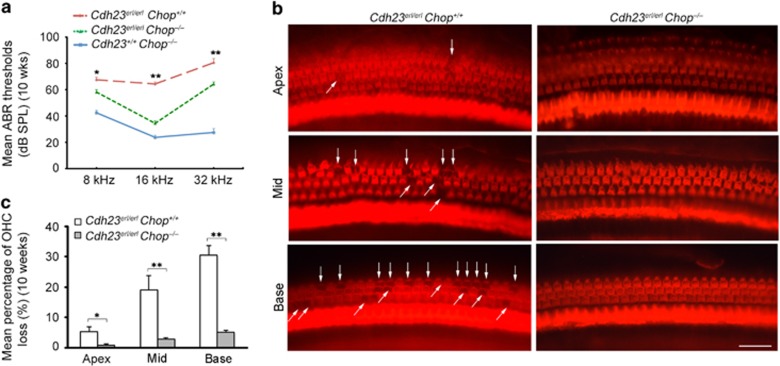
The hearing and OHC preservation in the Cdh23^*erl*/*erl*^*Chop*^−/−^ mice. (**a**) The ABR thresholds in the double-mutant Cdh23^*erl*/*erl*^*Chop*^−/−^ mice, *Chop*^−/−^ mice (Cdh23^*+*/*+*^*Chop*^−/−^) and *erl* mice (*Cdh23*^*erl/erl*^*Chop*^+/+^) at 8 , 16  and 32 k Hz stimuli at 10 weeks. The *Chop*^−/−^ mice showed normal hearing levels. The *erl* mice exhibited hearing impairment during all stimuli. The Cdh23^*erl*/*erl*^*Chop*^−/−^ double-mutant mice showed lower ABR thresholds than the *erl* mice during all stimuli. The error bars represent S.E.M. (**P<0.01, *P<0.05, *n*=8 mice, *t*-test). (**b**) The whole-mount preparations from the basal, middle and apical turns of the cochleae were stained for F-actin in the Cdh23^*erl*/*erl*^*Chop*^−/−^ mice and *erl* mice at 10 weeks. The *erl* mice showed some OHC losses in the apical turn and evident OHC loss in the middle and basal turns. No OHC loss was observed in the whole lengths of the Cdh23^*erl*/*erl*^*Chop*^−/−^ double-mutant cochleae. Scale bar, 20 *μ*m. (**c**) The mean percentages of OHC loss in the apical, middle and basal regions of the cochleae in the Cdh23^*erl*/*erl*^*Chop*^−/−^ and *erl* mice at 10 weeks. The *erl* mice showed significantly higher percentages of OHC loss than the Cdh23^*erl*/*erl*^*Chop*^−/−^ mice. The error bars represent S.E.M. (**P<0.01, *P<0.05, *n*=5 mice, *t*-test)

To further confirm the involvement of *Chop* in ER stress-induced apoptosis in *erl* mice, we used double-mutant mice containing the *erl* mutation in the *Cdh*23 gene and a disruption in the *Chop* gene. The inbred mice were obtained by crossing *Chop*^–/–^ mice with *Cdh23*^*erl/erl*^ mice. Nine genotypes of mice sharing the same genetic background (C57BL/6) were generated: *Cdh23*^+/+^*Chop*^+/+^; *Cdh23*^+/+^ *Chop*^+/–^; *Cdh23*^+/+^*Chop*^*–/–*^; *Cdh23*^+/erl^*Chop*^+/+^; *Cdh23*^+/erl^ *Chop*^+/–^; *Cdh23*^+/erl^ *Chop*^*–/–*^; *Cdh23*^*erl/erl*^ *Chop*^+/+^; *Cdh23*^*erl/erl*^ *Chop*^+/–^; and *Cdh23*^*erl/erl*^ *Chop*^*–/–*^. Supplementary Figure 1 shows the genotyping of the double-mutant *Cdh23*^*erl/erl*^ *Chop*^*–/–*^ mice. We measured the hearing and cytocochleograms of the *Cdh23*^*erl/erl*^*Chop*^*–/–*^; *erl* (*Cdh23*^*erl/erl*^ *Chop*^+/+^); and *Chop*^–/–^ (*Cdh23*^+/+^*Chop*^*–/–*^) mice. At 10 weeks, the auditory-evoked brainstem response (ABR) thresholds in the *Cdh23*^*erl/erl*^*Chop*^*–/–*^ double-mutant mice were significantly better than those in the *erl* mice (Figure 5a). The surface preparation performed at the same age revealed less loss of OHC in the *Cdh23*^*erl/erl*^*Chop*^*–/–*^ double-mutant mice in comparison with *Cdh23*^*erl/erl*^ mice. In the *erl* mice, several spots of OHC loss were exhibited in the basal and middle turns, and a few OHC losses were shown in the apical turns of the cochleae. However, in the *Cdh23*^*erl/erl*^*Chop*^*–/–*^ double-mutant mice, no OHC loss was found in the entire length of the cochleae (Figure 5b). The quantitative study showed that the mean percentage of OHC loss in the *Cdh23*^*erl/erl*^*Chop*^*–/–*^ mice was significantly lower than that in the *erl* mice (Figure 5c).

### Sal prevented hearing loss in *erl* mice

**Figure 6:**
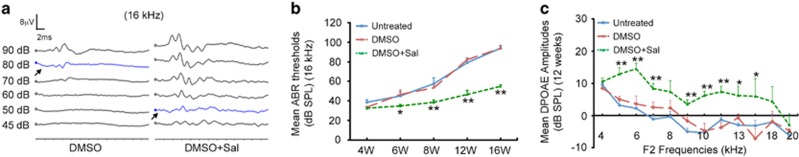
Sal improved hearing in the *erl* mice. (**a**) At 12 weeks, the vehicle-treated mice showed much higher ABR thresholds than the Sal-treated mice at 16-kHz stimuli. The colored lines and arrows represent threshold waveforms. (**b**) The 16-kHz-stimulated-ABR thresholds in Sal-treated mice were significantly lower than those in the DMSO and untreated mice from 4 (W) to 16 weeks. No significant difference was found between the untreated and DMSO groups. (**c**) The DPOAE amplitudes in the Sal-treated mice were much higher than in the untreated and DMSO-treated mice at 12 weeks. No significant difference was found between the untreated and DMSO groups. The error bars represent S.E.M. (**P<0.01, *P<0.05, *n*=5 per group, one-way ANOVA)

After receiving corresponding treatments, the *erl* mice in the test, vehicle and control groups underwent ABR and distortion product otoacoustic emission (DPOAE) testing at the same ages. The ABR thresholds in the test group were significantly lower than those in the vehicle and control groups in response to 16 (Figure 6a and b), 8 and 32 kHz tone-burst and click stimuli (Supplementary Figure 2). No significant difference was found between the vehicle and control groups. The otoprotective effect was further confirmed by the higher DPOAE amplitudes. At 8 weeks, the DPOAE amplitudes in the test group were significantly greater at high frequencies (Supplementary Figure 3a). At 12 weeks, the test group showed much higher DPOAE amplitudes at almost all frequencies (Figure 6c). At 16 weeks, mice in all three groups showed lower DPOAE amplitudes, whereas the test group's amplitudes remained higher at low frequencies (Supplementary Figure 3b).

### Sal protected against OHC death in *erl* mice

**Figure 7:**
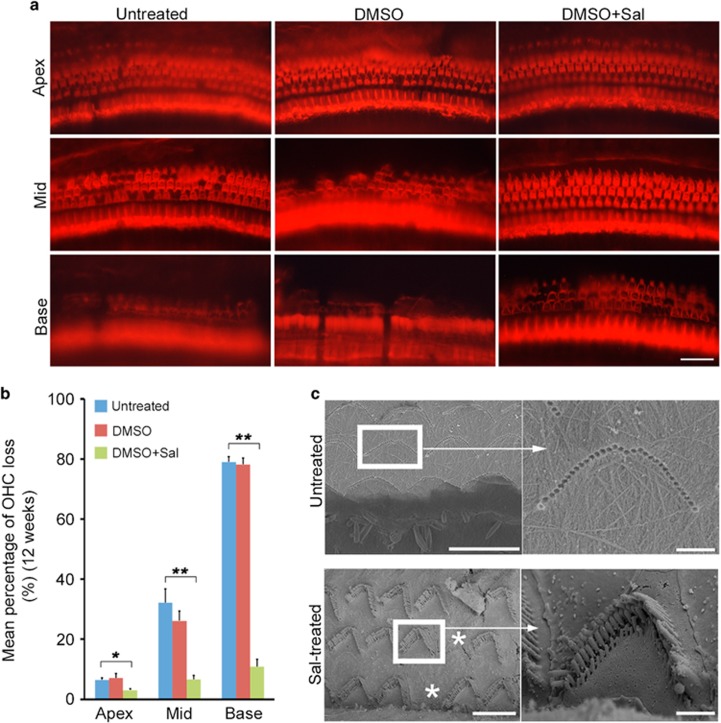
Sal prevented OHC loss in the *erl* mice. (**a**) The whole-mount preparations from the basal, middle and apical turns of the cochleae in the untreated, DMSO-treated and Sal-treated mice at 12 weeks. In the vehicle and control groups, OHC loss was observed in the basal and middle turns, and such losses were more pronounced in the basal turn. In the apical turn, the vehicle and control groups showed disorganized OHC patterns but almost no cell loss. The test group showed small amounts of OHC loss in the basal and middle turns and a normal arrangement in the apical turn. Scale bar, 20 *μ*m. (**b**) The mean percentages of OHC loss in the apical, middle and basal regions of the cochleae in the Sal-treated mice were significantly lower than in the untreated and DMSO-treated mice at 12 weeks. No significant differences were observed between the DMSO and untreated groups. The error bars represent S.E.M. (**P<0.01, *P<0.05, *n*=4 per group, one-way ANOVA). (**c**) The OHC morphology and subcellular structures by scanning electron microscope from the basal turns of the cochleae of untreated and Sal-treated *erl* mice at 12 weeks. The untreated mice showed almost total OHC loss and mild inner-hair cell loss in the basal turn. The Sal-treated mice showed little OHC loss and an almost normal arrangement of hair bundles. Stars mark the OHC losses. Scale bars, 10 *μ*m (left); 1 *μ*m (right)

A cochlear surface preparation was performed on *erl* mice in the test, vehicle and control groups at 12 weeks. OHC impairment was obvious in the vehicle and control groups, while OHC loss was very minimal in the test group (Figure 7a). In the vehicle and control groups, several substantial contiguous spots of OHC loss were observed in the basal and middle turns, and some OHC loss was seen in the apex turns. In contrast, the mice in the test group showed only small amounts of OHC loss in the basal turn and rare OHC loss in the middle turn. The mean percentage of OHC loss in the test group was significantly lower than that in the vehicle and control groups (Figure 7b). No significant difference was found between the vehicle and control groups.

A scanning electron microscope was used to analyze OHC morphology and subcellular structures at 12 weeks (Figure 7c). The images showed that untreated *erl* mice exhibited almost total OHC loss and no detectable subcellular structure of hair bundles in the basal turn. In contrast, the Sal-treated mice showed small amounts of OHC loss and approximate normal arrangements of hair bundles.

### Sal suppressed ER stress-induced apoptosis in *erl* mice

**Figure 8:**
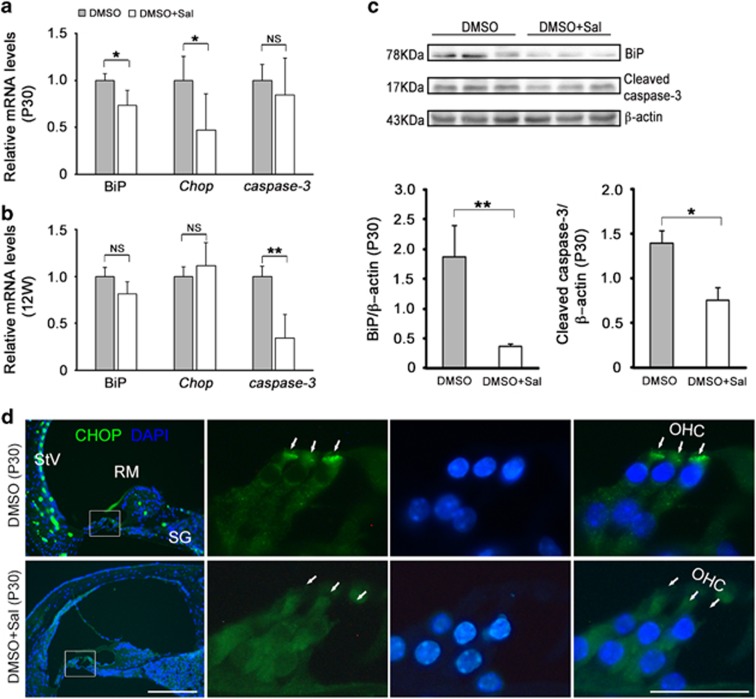
Sal downregulated ER stress and apoptosis-related genes and proteins. (**a**) *BiP* and *Chop* mRNA were downregulated in the Sal-treated mice at P30. However, no significant difference was found in the *Caspase-3* mRNA levels (**P<0.01, *P<0.05, NS, not significant, *n*=3, *t*-test). (**b**) At 12 weeks, the *Caspase-3* mRNA levels were remarkably downregulated in the Sal-treated mice compared with DMSO-treated mice; however, no significant differences were found in the *BiP* and *Chop* mRNA levels (**P<0.01, *P<0.05, NS, not significant, *n*=3, *t*-test). (**c**) The BiP and cleaved caspase-3 protein levels in the cochleae from the Sal-treated mice were much lower than those from the DMSO-treated mice at P30. Significant differences were found between the DMSO and Sal-treated mice in their quantities of BiP and cleaved caspase-3. The error bars represent S.E.M. (**P<0.01, *P<0.05, NS, not significant, *n*=3, *t*-test). (**d**) CHOP signals were more strongly detected in the OHCs, SG and StV of the DMSO-treated mice, showing high concentration in the perinuclear region in between nucleus and hair bundles of OHCs but attenuated by Sal (*n*=3 mice per group). Scale bars, 50 *μ*m (left); 20 *μ*m (right)

ER stress and apoptosis-related genes and proteins were downregulated after the Sal treatments. The mRNA expression levels of *BiP*, *Chop, caspase-3* and *caspase-12* genes were tested at P30 and 12 weeks. The results showed that *Bip* and *Chop* decreased in the Sal group versus the vehicle group at P30, and *caspase-3* was downregulated at 12 weeks (Figures 8a and b). However, no significant difference was found in the *caspase-12* gene expression (Supplementary Figure 4). BiP and cleaved caspase-3 protein extracted from the *erl* mouse cochleae in the vehicle and test groups were measured by Western blot at P30. The results showed that BiP and cleaved caspase-3 were much less abundant in the Sal-treated cochleae than in the vehicle-treated cochleae (Figures 8c). Furthermore, CHOP was immunolabeled in the vehicle and test groups at P30. The results showed that the CHOP signals in the OHCs, SG and StV in DMSO-treated cochleae were distinctly weakened by Sal treatment, and the most significant difference in CHOP expression was seen in the perinuclear regions of the OHCs (Figures 8d).

## Discussion

As a novel mutation of the *Cdh*23 gene, the *erl* mutant mice exhibited the postnatal onset of hearing loss starting at P27, which progressed to total deafness at ~P100. This is considered to be an animal model for human DFNB12.^10^ Given their time window from initial hearing-loss initiation to total deafness, these mutant mice are ideal tools for testing new otoprotective drugs. We previously revealed that *erl* mice treated with erythropoietin and Z-VAD-FMK could be significantly protected against OHC death and rescued from hearing loss through the blocking of the apoptosis pathway in *erl* mice.^10, 16^ In the current study, the upstream apoptosis response in the *erl* cochleae was discovered to be relevant to ER stress and the UPR.

Under the quality-control surveillance of the ER, only correctly folded proteins can be transferred by the Golgi complex and sent to their final destinations, while unfolded or misfolded proteins are retained in the ER and ultimately degraded by the ubiquitin-dependent proteolytic pathway or autophagy.^17, 18^ The accumulation and aggregation of unfolded or misfolded proteins in the ER lumen increase ER loading and disrupt ER homeostasis, ultimately leading to ER stress.^19, 20^ Under mild-to-moderate stimulation, ER stress is an adaptive and restorative response that leads to cell adaptation. In contrast, if the stress is beyond the ER's adaptive capacity, the protective signaling will switch to pro-apoptotic responses. ER stress has been linked to the pathogenesis of neurodegenerative diseases, such as Alzheimer's disease and Parkinson's disease, as well as to the pathogenesis of cell death, such as renal tubule lesions in diabetes.^21, 22, 23^ In zebrafish mutants, the defective CDH23 proteins in hair cells failed to be transferred and contributed to the formation of Usher protein complexes, therefore leading to ER stress.^15^ Taken together, these results offered us new insight into the pathological mechanisms of and potential therapies for sensorineural deafness. Using an animal model for mammals, we questioned whether similar protein-folding defects and the activation of ER stress happened in mice with the *Cdh*23 mutation.

We provided evidence that part of the ERL-CDH23 protein exhibited abnormal localization in the OHC (from the stereocilia to the nucleus, Figure 1a), failing to reach the top of hair bundles and thus leading to reduced migration to the tip links. Moreover, the ERL-CDH23 protein in the subapical region of the OHC was co-localized with ER chaperone BiP (Figures 1b and c). The BiP protein was found to be highly expressed in OHCs, SG and StV in the *erl* cochleae at P6 and P12, and the BiP mRNA was upregulated at P12 but downregulated at P30 (Figure 2). Considering the UPR pathway, these results suggest that BiP was dissociated with the ER transmembrane receptor and then combined with defective CDH23 at the early stage of this response. Downstream of this pathway, eIF2α phosphorylation was detected in the *erl* cochleae (Figure 3), and the *Chop* mRNA and protein were upregulated at P12 and P30 (Figure 4). These results indicate that the PERK arm of the UPR was activated. CHOP was a key mediator when the ER stress-induced UPR signaling progressed to apoptosis.^24, 25^ The results of the auditory test, surface preparation and OHC counting in our double-mutant *Cdh23*^*erl*/*erl*^ *Chop*^−/−^ mice further confirm that the deletion of the *Chop* gene could partly protect hearing and preserve the OHC in mice with the *Cdh23*^*erl/erl*^ mutation (Figure 5). Taking these data together with the previous study on the induction of apoptosis-related genes in the *erl* mouse cochleae,^10^ we suggest that apoptosis is an event downstream of the induction of the UPR, with the effector PERK signaling pathway being a contributor. Thus, we selected the PERK arm of the UPR as a therapeutic target.

It is interesting to note that CHOP does not present nuclear localization in OHCs, as expected for a transcription factor (Figures 4 and 8). However, CHOP does not have a nuclear localization signal. Its interaction with other stress-induced transcription factors mediates its nuclear localization.^26^ It is therefore possible that in OHCs, CHOP is excluded from the nucleus due to the absence of interacting transcription factor partners. It was also reported that cells with cytoplasmic and nuclear localized CHOP gave distinct gene expression profiles. It was shown that that cytoplasmic CHOP inhibited the migration, while nuclear CHOP caused a G1 cell cycle arrest.^27^ In this *erl* model, particularly in OHCs, CHOP is localized in the perinuclear regions in between the nuclei and hair bundles of the OHCs. An OHC develops a polarized bundle of stereocilia that is important for mechanotransduction.^28^ Many hair bundle proteins migrate toward the hair bundle; thus, we speculate that the cytoplasmic CHOP may regulate the expression of protein migration-related genes. Further study is warranted on CHOP's role(s) aside from inducing apoptosis on OHC degeneration and functionality.

Sal is a small molecular compound (480 Da) that prevents cell death from ER stress-induced apoptosis by selectively inhibiting the dephosphorylation of eIF2α.^29^ Studies show that Sal resists ER stress-induced cell death, improves cell survival and delays disease processes.^30, 31, 32, 33, 34^ Our hearing tests confirmed our hypothesis that Sal could protect hearing by inhibiting ER stress-induced hair cell apoptosis in the *erl* mice. In a previous work, we reported that progressive hearing loss started at P27 in *erl* mice.^10^ In this study, as early as 4 weeks postnatal, the 32-kHz-stimulus-evoked ABR thresholds in the test group were 10 dB better than in the control and vehicle groups (Supplementary Figure 1b). At all subsequent time points, the Sal-treated mice showed better ABR thresholds under all stimuli. Referring to the correspondence between high and low frequencies and to the basal-apical gradient of the basilar membrane,^35, 36^ the auditory tests indicate that the OHC death specifically started in the basal turns and then spread through the entire cochleae, which could be remedied by Sal treatment. Our previous studies targeting anti-apoptosis treatments also showed otoprotective effects,^10, 16^ but Sal showed an extended duration (up to 16 weeks). In our histological examination, we observed substantial contiguous and isolated OHC losses in the vehicle and control groups; in contrast, the Sal-treated mice showed only small amounts of OHC loss (Figure 7). This result provides a good correlation between our anatomical observations and functional tests.

After the Sal treatment, the upregulated *Bip* and *Chop* mRNA were decreased at P30, and the *caspase-3* was decreased at 12 weeks (Figure 8a), indicating that *Bip* and *Chop* were affected by Sal treatment at an early age and that the *caspase-3* mRNA was influenced later. However, the cleaved caspase-3 proteins (detected by western blot) were found to be decreased by Sal at P30. Our cleaved caspase-3 antibody (Asp175) was able to recognize the large fragment (17 kDa) of activated caspase-3 resulting from the cleavage adjacent to Asp175, but it was incapable of detecting the full-length caspase-3 protein. These results suggest that the caspase-3 cleavage was blocked at P30 by Sal treatment. The *caspase-12* mRNA remained unchanged at P30 and 12 weeks (Supplementary Figure 4), indicating that *caspase-12* might not be involved in the feedback of the Sal treatment. The BiP proteins (detected by Western blot) and CHOP proteins (detected by immunostaining) were also downregulated after Sal treatment (Figure 8). These results, together with the previous data,^10^ suggest that the defective CDH23 accumulated in the ER and then increased the ER loading and destroyed ER homeostasis, leading to the dissociation of the BiP from PERK. The downstream upregulated CHOP led to caspase activation and apoptosis. If the adaptive UPR reduces the protein load in the ER, then the restoration of protein synthesis will promote cell survival. Otherwise, if protein synthesis overrides the restoration of proteostasis, cell death will be triggered.^37^ Sal inhibits the dephosphorylation of eIF2α, which inhibits protein synthesis when it is phosphorylated by PERK. This increased phosphorylation of eIF2α limits protein synthesis (including that of CDH23 and CHOP), relieves ER loading, and promotes cell survival. Potential mechanisms of the pro-apoptotic function of uncontrolled protein synthesis during ER stress have recently been described.^37, 38^

As an animal model of DFNB12, the *erl* mice with Sal treatment began at an early age (starting from P7) and showed significant hearing protection for a long duration (up to 16 weeks). By considering the signaling pathway of ER stress-induced apoptosis, we suggest that therapies targeting this specific signaling pathway could prevent hair cell loss at an early stage and achieve more effective and sustained results. USH1D patients who carry different mutations of the same gene (*Cdh23*) as DFNB12 exhibit congenital deafness and postnatal-onset blindness. Thus, we assume that early treatment with Sal might prevent ophthalmic dysfunction in Usher syndrome, thus highlighting Sal's application as a therapeutic agent in the treatment of animal models for Usher syndrome.

## Conclusion

In summary, this study is the first to consider ER stress-induced hair cell apoptosis as having a key role in the hearing loss and hair cell death of mice with the *Cdh23* mutation. Protein synthesis and related protein-folding processes are early molecular events in ER stress and apoptosis. Thus, protein-folding errors triggered by genetic mutations should be considered the earliest therapeutic target. Many Food and Drug Admission (FDA)-approved anti-ER stress drugs are presently available on the market and thus can be repurposed for otoprotection.

## Materials and Methods

### Experimental design

This study aimed at assessing ER stress-induced hair cell apoptosis in the mechanism of hearing loss and hair cell death in *Cdh23*^*erl/erl*^ mutant mice. The measurements of ER stress indicators were performed between *Cdh23*^*erl/erl*^ mutant mice, control B6 mice, as well as the *Chop* gene knockout (C57BL/6 background) mice. The ER stress inhibitor Salubrinal (Sal) was systematic given in *Cdh23*^*erl/erl*^ mutant mice by intraperitoneal injection. The hearing, OHC reservation and ER stress indicators were tested between Sal-treated, vehicle-treated and untreated *Cdh23*^*erl/erl*^ mice.

### Mice

All of the procedures involving mice were approved by the Animal Research Committee of Case Western Reserve University School of Medicine (protocol R01DC009246). All of the mice were experimented upon in the same environment and bright-dark circle. The *erl* mice were first housed in the Jackson Laboratory (Bar Harbor, ME, USA) before this mutant strain was relocated to Case Western Reserve University. Mice with the *Chop* gene knockout (C57BL/6 background) were introduced from the Jackson Laboratory (stock number 005530). The homozygous *erl* mice (*Cdh23*^*erl/erl*^) were crossed with the homozygous *Chop* gene knockout mutants (*Chop*^−/−^), generating F1 with the genotype of *Cdh23*^+/erl^ *Chop*^+/–^. Then, the F1 (*Cdh23*^+/erl^ *Chop*^+/–^) was crossed with F1 (*Cdh23*^+/erl^ *Chop*^+/–^) to generate nine genotypes of mice: *Cdh23*^+/+^*Chop*^+/+^; *Cdh23*^+/+^ *Chop*^+/–^; *Cdh23*^+/+^*Chop*^*–/–*^; *Cdh23*^+/erl^*Chop*^+/+^; *Cdh23*^+/erl^ *Chop*^+/–^; *Cdh23*^+/erl^ *Chop*^*–/–*^; *Cdh23*^*erl/erl*^ *Chop*^+/+^; *Cdh23*^*erl/erl*^ *Chop*^+/–^; and *Cdh23*^*erl/erl*^ *Chop*^*–/–*^. Then, we selected the homozygous *Cdh23*^*erl/erl*^ *Chop*^*–/–*^, which were intercrossed and maintained as an inbred mouse line. Because the *Chop* gene knockout and the *erl* mutation mice are both come from the C57BL/6 background, we chose *Cdh23*^*erl/erl*^ *Chop*^*–/–*^, *Cdh23*^*erl/erl*^ *Chop*^+/+^(*erl* mice) and *Cdh23*^+/+^*Chop*^*–/–*^(*Chop*^*–/–*^ mice) for our experiments.

The genotyping for *erl* mice was described previously.^10^ The genotype for *Chop*^*–/–*^ was determined by semi-quantitative PCR through the use of the following primers: oIMR3884-5′-ATGCCCTTACCTATCGTG-3′ (common); oIMR3885 - 5′-AACGCCAGGGTTTTC CCAGTCA-3′ (mutant reverse); and oIMR3886 - 5′-GCAGGGTCAAGAGTAGTG-3′ (wild-type reverse). The thermal cycle reaction was performed as follows: 94 °C for 3 min, followed by 35 cycles at 94 °C for 30 s, 61 °C for 1 min, 72 °C for 1 min and 72 °C for 2 min. The wild-type showed one band (544 bp), whereas the heterozygotes showed two bands (320 and 544 bp) and the mutants showed one band (320 bp). The genotype for *Cdh23*^*erl/erl*^ *Chop*^*–/–*^ was determined by *Cdh23*^*erl/erl*^ genotyping and *Chop*^*–/–*^ genotyping.

### Sal treatment

The *erl* mice received different treatments by intraperitoneal injection. In total, 70 *erl* mice were divided into three groups with either gender: a test group (treated with Sal, 0.5 mg kg^−1^, R&D ng Cat# 2347), a vehicle group (treated with an equal volume of dimethylsulfoxide, DMSO), and a control group (untreated). All of the treatments started from postnatal (P) day 7, with subsequent injections occurring every other day for the first 12 weeks, every 3 days for 2 additional weeks, and then once every week for the duration of the experiments. The Sal dosage was selected from two sets of preliminary experiments, which showed it to be safe and hearing protective. The starting age of treatment was selected to prevent apoptotic gene upregulation in *erl* mice detected in our previous work.^10^

### PCR

The cochleae were quickly isolated, and then TRIzol (Invitrogen, Eugene, OR, USA) was used for total RNA extraction. The Super Script III First-Strand Synthesis System (Invitrogen) was used to synthesize cDNA. Quantitative PCR was performed with the reagent of SYBR Green FAST Mastermix (QIAGEN, Germantown, MD, USA) on an ABI 7300 Sequence Detection System (Foster City, CA, USA). Primer sequences are listed in Table. S1. The cycle reaction was performed as follows: 95 °C for 10 min, 40 cycles of 95 °C for 15 s and 60 °C for 1 min, and finally a dissociation curve of 95 °C for 15 s and 60 °C for 15 s. The gene expression was calculated relative to the housekeeping gene GAPDH and then analyzed using the 2^−ΔΔCT^ method. Semi-quantitative PCR was performed using the DNA Engine (Bio-Rad, Hercules, CA, USA) with the cycle reaction described above.

### Western blotting

The cochleae were lysed using ice-cold RIPA buffer. Equal amounts of protein were subjected to SDS-PAGE and transferred onto the PVDF (Bio-Rad) membrane. Afterward, the PVDF membrane was blocked for 1 h in 5% ECL prime blocking agent and then incubated overnight at 4 °C with 1:1000 diluted primary antibodies: anti-BiP (Cell Signaling 3177, Danvers, MA, USA), anti-caspase-3 (Cell Signaling 9661) and anti-β-actin (Santa Cruz sc-130657). After washing with TBST, the membrane was incubated in secondary antibodies (1:5000, goat anti-rabbit IgG, Santa Cruz sc-2054). Protein bands were visualized using the chemiluminescence-emanating ChemiDoc MPImaging System (Bio-Rad).

### Immunofluorescence staining

The inner ears were fixed in 4% paraformaldehyde (PFA) overnight and then decalcified in 10% ethylenediaminetetraacetic acid (EDTA). After being dehydrated in sucrose and embedded in tissue OCT freeze medium at −20 °C, 6 *μ*m continuous sections were cut. These sections were permeabilized in 0.5% Triton X-100 for 30 min, blocked in 3% BSA for 2 h at room temperature, and then incubated overnight at 4 °C with 1:200 diluted primary antibodies: anti-CDH23 (Santa Cruz sc-26338), anti-BiP (Cell Signaling 3177), anti-CHOP (Santa Cruz sc-575), anti-eIF2a (Cell Signaling 9722) and anti-phospho-eIF2a (Cell Signaling 3597). Secondary antibodies with 1:400 dilution (donkey anti-rabbit IgG, Invitrogen A31573; rabbit anti-goat IgG, Invitrogen A11078) were added for 1 h; the sections were then counter-stained with DAPI. The negative controls (with no primary antibodies added) showed no staining.

### ABR and DPOAE testing

A computer-aided evoked potential system (Intelligent Hearing Systems, the Smart-EP software 3.30, Miami, FL, USA) was used as previously published.^39^ Briefly, the mice were anesthetized, and their body temperature was maintained at 37 °C. Sub-dermal needle electrodes were used. The recording electrode was inserted at the vertex of the skull; the ground electrode was inserted in the apex of the nose; and the reference electrodes were fixed near each ear. Click and 8, 16 and 32 kHz tone bursts were channeled through an inserted earphone. The ABR threshold was identified as the lowest stimulus level at which clear and repeatable waveforms were recognized (Figure 2a). The DPOAE measurement was conducted for pure tones at frequencies ranging from 4.4 to 20.3 kHz by using the Intelligent Hearing System (Smart EP 3.30 Software). Frequencies were acquired with the F2:F1 ratio of 1.22 and with primary stimulus of 65/55 dB SPL.

### Surface preparation and hair cell counting

As we previously described,^40^ the temporal bones were fixed in 4% PFA, and the basilar membrane was then carefully dissected and cut into three separate fragments: apical, middle and basal turn. The fragments were permeabilized in 2% Triton X-100, stained for F-actin with phalloidin (Invitrogen), and observed with a fluorescence microscope (Leica DM4500 B). Hair cells were counted as being present if cell bodies and the V-shaped hair bundles were intact.

### Confocal 3D imaging

To determine CDH23 localization in OHCs, whole-mount immunostaining of inner ear basilar membranes from 4-day-old mice was conducted using primary antibody anti-CDH23 (Santa Cruz, sc-26338), secondary rabbit anti-goat IgG, Invitrogen A11078, Alexa 488 (1:1000 dilution), then counter-stained with DAPI. Confocal images were captured using a Leica (Germany) SP8 confocal microscope with a × 63/1.4 NA oil objective and a 488 nm laser-line. Imaging stacks acquired with voxel-size 512 × 512 × 0.045 *μ*m^3^ and 1024 × 1024 × 0.027 *μ*m^3^, respectively depending on the sizes of OHCs, All deconvolutions were processed using Leica Application Suite X (LAS X, LASX) software.

### Scanning electron microscope

The mice were fixed with 2.5% glutaraldehyde, and then the whole cochleae were dissected. The bony capsule, spiral ligament, and Reissner's membrane were carefully removed to expose the whole organ of Corti. Afterwards, the specimens were fixed in 1% osmium tetroxide (OsO4) for 1 h three times and in 1% thiocarbohydrazide (TCH) for 1 h twice (the OTOTO technique). The specimens were dehydrated in a gradient ethanol series, critical-point dried using CO_2_, and finally coated in palladium. Then, the samples were viewed under a high-resolution scanning electron microscope (FEI Helios NanoLab 650, Germany).

### Statistical analysis

The data are presented as mean±S.E.M. The analysis was performed using the SPSS 18.0 software. The data were statistically analyzed using one-way ANOVA and Student's *t*-tests. *P*-values<0.05 were considered significant.
