# Supplementary material for: ER stress inhibitor attenuates hearing loss and hair cell death in Cdh23erl/erl mutant mice
Source: Cell Death Dis. 2016 Nov 24;7(11):e2485–. doi: 10.1038/cddis.2016.386 (PMC5260868; doi:10.1038/cddis.2016.386)
Supplement: Supplementary Material [file cddis2016386x1.docx]

**Supplementary Materials**

**ER stress inhibitor attenuates hearing loss and hair cell death**

**in Cdh23*^erl/erl^* mutant mice**

Juan Hu, Bo Li, Luke Apisa, Heping Yu, Shami Entenman, Min Xu, Ruben Stepanyan, Bo-Jhih Guan, Ulrich Müller, Maria Hatzoglou, Qing Yin Zheng

**
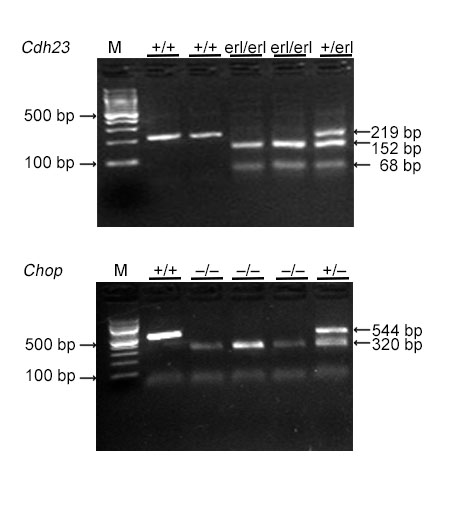
**

**Fig. S1** Genotyping of double-mutant mice. The genotyping of *Cdh23* was identified from PCR products with primers ex3m-F and ex3m-R, and digestion with BstN I restriction endonuclease (upper panel). The wild type showed one band (219 bp). The homozygous *erl* mutants showed two fragments (152 and 68 bp), and the heterozygote showed three bands (219, 152, and 68 bp). The *Chop* genotype was determined by PCR products with primers oIMR3884, oIMR3885, and oIMR3886. The wild type showed one band (544 bp). The heterozygote showed two bands (320 bp and 544 bp), and the homozygous *Chop*^–/–^ mutants showed one band (320 bp).


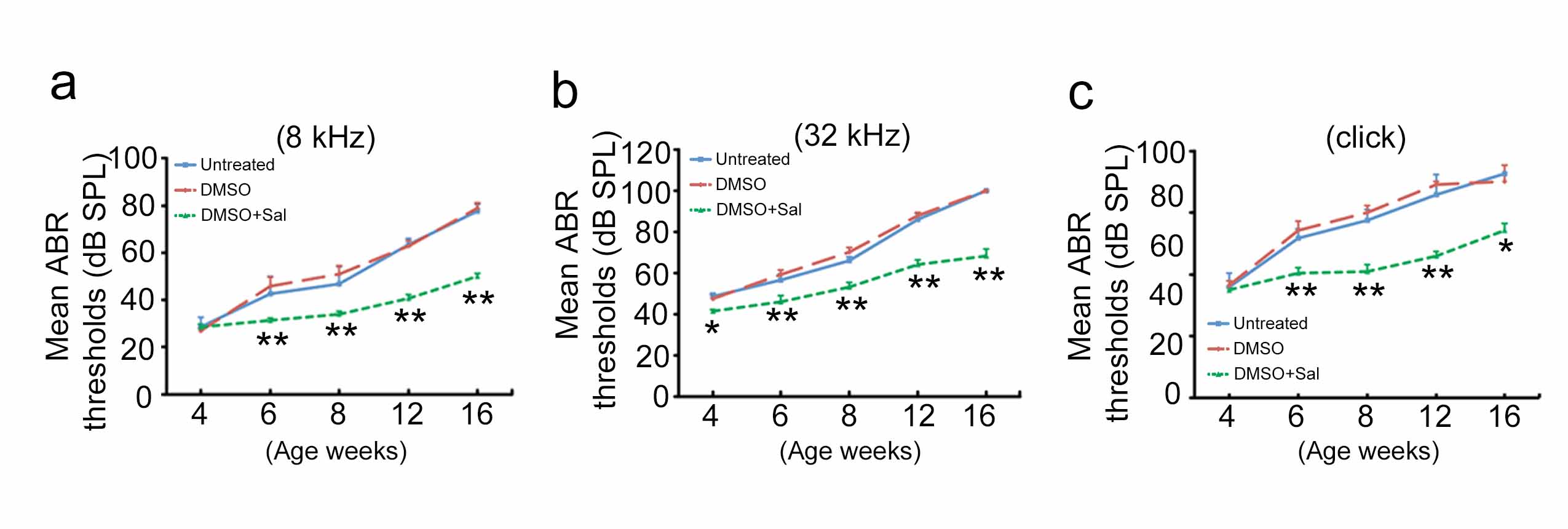


**Fig. S 2** Sal reduced the ABR thresholds in the *erl* mice. (a, c) The ABR thresholds (evoked by 8kHz tone burst and click) in the Sal-treated mice were significantly lower than that those in the DMSO and untreated groups from the age of 6 weeks, indicating better hearing in the Sal-treated mice. No significant difference was found between the untreated and DMSO groups. (b) As early as 4 weeks of age, the 32kHz-stimulus-evoked ABR thresholds in the Sal group were 10dB better than in the untreated and vehicle groups. At all subsequent time points (≥6 weeks), the Sal-treated mice showed better ABR thresholds evoked by all stimuli. No significant differences were found between the untreated and DMSO groups. The numbers of untreated, DMSO, and Sal mice were 10, 10, and 14 at 4 weeks (W); 6, 6, and 10 at 6 weeks and 8 weeks; and 5, 5, and 6 at 12 and 16 weeks, respectively. The error bars represent SEM.


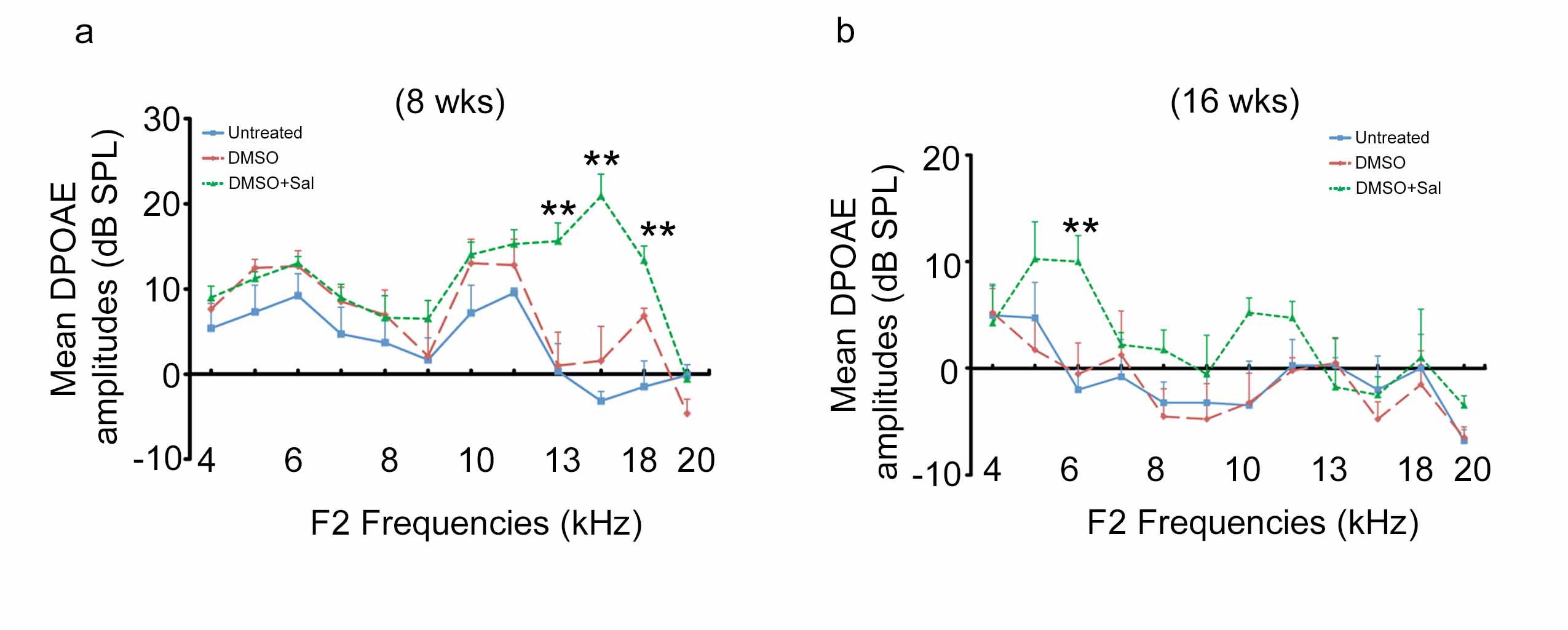


**Fig. S 3** Sal increased the DPOAE amplitudes in the *erl* mice. The DPOAE amplitudes in the Sal-treated mice were much higher than in the untreated and DMSO-treated mice at high frequencies at 8 weeks (a) and at low frequencies at 16 weeks (b), indicating OHC functional protection in the Sal-treated mice. No significant difference was found between the untreated and DMSO groups. The numbers of untreated, DMSO, and Sal mice was 10, 10, and 14 at 4 weeks (W); 6, 6, and 10 at 6 and 8 weeks; and 5, 5, and 6 at 12 and 16 weeks, respectively. The error bars represent SEM.


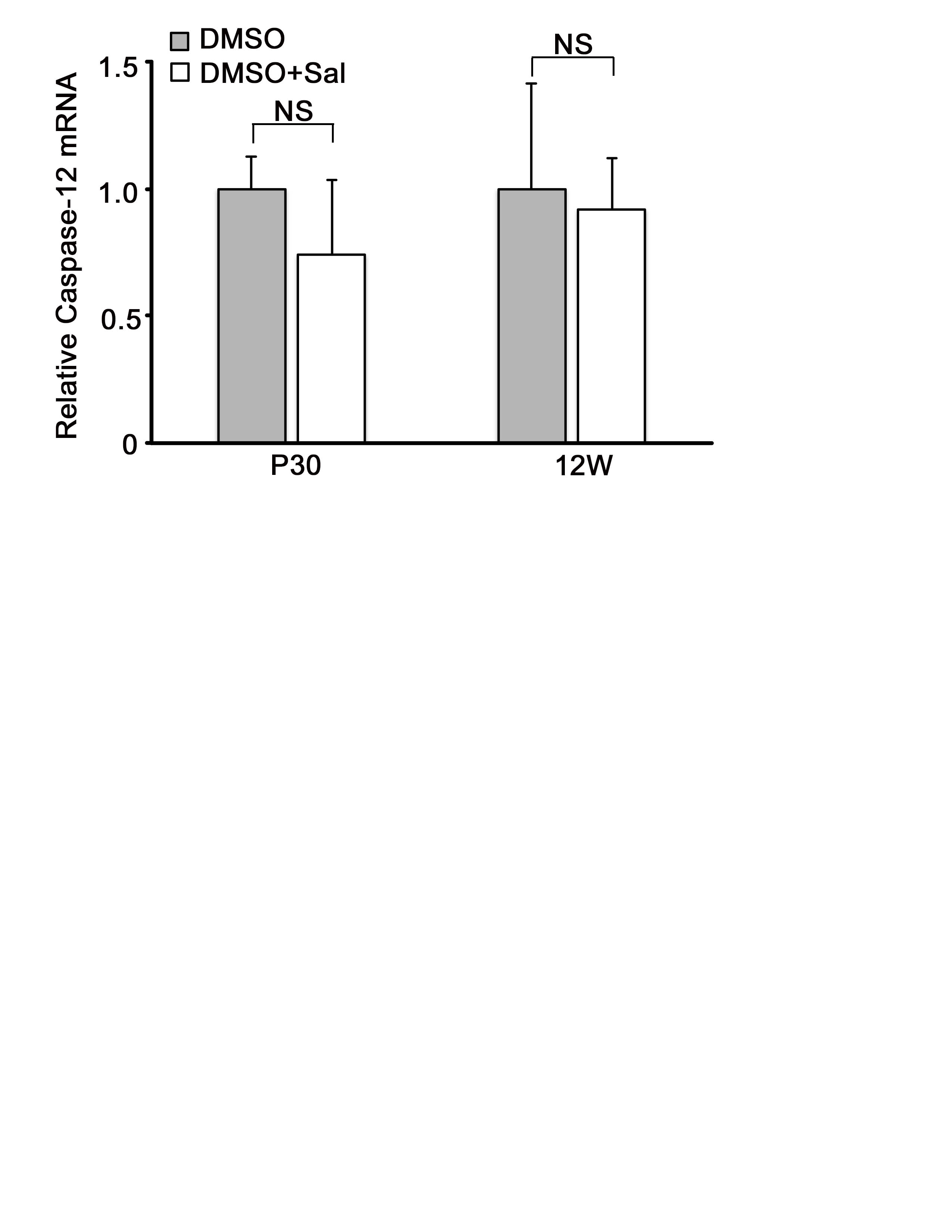


**Fig. S 4** No significant differences in *caspase-12* mRNA levels were found between the Sal-treated and DMSO-treated mice at P30 and 12 weeks. The error bars represent SEM.

**Table. S1.** Primer Sequences

| primers | sequences |
| --- | --- |
| ex3m F | CAGGTTCTTCTGTGACCCAGC |
| ex3m R | CCA AAAGATGGTGAGCCACCT |
| oIMR3884 | ATGCCCTTACCTATCGTG |
| oIMR3885 | AACGCCAGGGTTTTCCCAGTC |
| oIMR3886 | GCAGGGTCAAGAGTAGTG |
| BIP F | ACTTGGGGACCACCTATTCCT |
| BIP R | ATCGCCAATCAGACGCTCC |
| Chop F | CTGGAAGCCTGGTATGAGGAT |
| Chop R | CAGGGTCAAGAGTAGTGAAGGT |
| Caspase-3 F | ATGGAGAACAACAAAACCTCAGT |
| Caspase-3 R | TTGCTCCCATGTATGGTCTTTAC |
| Caspase-8 F | ATGGCGGAACTGTGTGACTCG |
| Caspase-8 R | GTCACCGTGGGATAGGATACAGCA |
| Caspase-9 F | CCTAGTGAGCGAGCTGCAAG |
| Caspase-9 R | ACCGCTTTGCAAGAGTGAAG |
| Caspase-12 F | AGACAGAGTTAATGCAGTTTGCT |
| Caspase-12 R | TTCACCCCACAGATTCCTTCC |
| XBP-1 F | ACACGCTTGGGAATGGACAC |
| XBP-1 R | CCATGGGAAGATGTTCTGGG |
| Gapdh F | AGGTCGGTGTGAACGGATTTG |
| Gapdh R | TGTAGACCATGTAGTTGAGGTCA |
